# Ocular coloboma combined with cleft lip and palate: a case report

**DOI:** 10.1186/s12886-020-01696-3

**Published:** 2020-10-19

**Authors:** Yung Ju Yoo, Sang Beom Han, Hee Kyung Yang, Jeong-Min Hwang

**Affiliations:** 1 Department of Ophthalmology, Kangwon National University Hospital, Kangwon National University School of Medicine, 156 Baengnyeong-ro, Chuncheon, 24289 South Korea; 2 Department of Ophthalmology, Seoul National University College of Medicine, Seoul National University Bundang Hospital, Seongnam, South Korea

**Keywords:** Coloboma, Optic fissure closure, Cleft lip, Cleft palate, Case report

## Abstract

**Background:**

Ocular coloboma is an excavation of ocular structures that occurs due to abnormal fusion of the embryonic optic fissure. Further, cleft lip/palate (CL/P), a congenital midline abnormality, is caused by a defect in the fusion of the frontonasal, maxillary, and mandibular prominences. No study has reported the association between these two phenotypes in the absence of other systemic abnormalities. We present a case of ocular coloboma along with CL/P and without other neurological abnormalities.

**Case presentation:**

A 5-year-old Asian boy presented with decreased visual acuity in his right eye. Physical examination revealed no abnormal findings except CL/P, which was surgically corrected at the age of 9 months. Best-corrected visual acuity was 20/60 in the right eye and 20/25 in the left eye. Anterior segment examination revealed iris coloboma in the inferior quadrant of his right eye as well as a large inferonasal optic disc and chorioretinal coloboma in the same eye. He was prescribed glasses based on his cycloplegic refractive errors and part-time occlusion of the left eye was recommended. After 3 months, best-corrected visual acuity improved to 20/30 in the right eye.

**Conclusion:**

The association of ocular coloboma should be kept in mind when encountering a patient with CL/P without other neurological or systemic abnormalities.

## Background

Ocular coloboma is characterized by the absence of the iris, lens, retina, choroid, and/or optic nerve in the inferonasal quadrant of the eye and has variable phenotypes [1]. It occurs due to abnormal fusion of the embryonic optic fissure, which is normally completed by the sixth week of gestation [2, 3]. Several studies have demonstrated that thalidomide, maternal vitamin A deficiency, toxoplasmosis, and cytomegalovirus infection can result in this condition [1]. Ocular coloboma may be associated with other developmental abnormalities, and there is a rare case of branchio-oculo-facial syndrome in which ocular coloboma, CL/P, branchial arches, and facial malformation are combined [4]. Although rarely reported, CL and choroidal coloboma can occur along with hypothalamo-pituitary dysfunction [5]. To the best of our knowledge, no study has reported ocular coloboma along with CL/P in a patient without other neurological abnormalities. Here, we report a recently encountered case of unilateral ocular coloboma with CL/P and without other systemic abnormalities.

## Case presentation

**Fig. 1:**
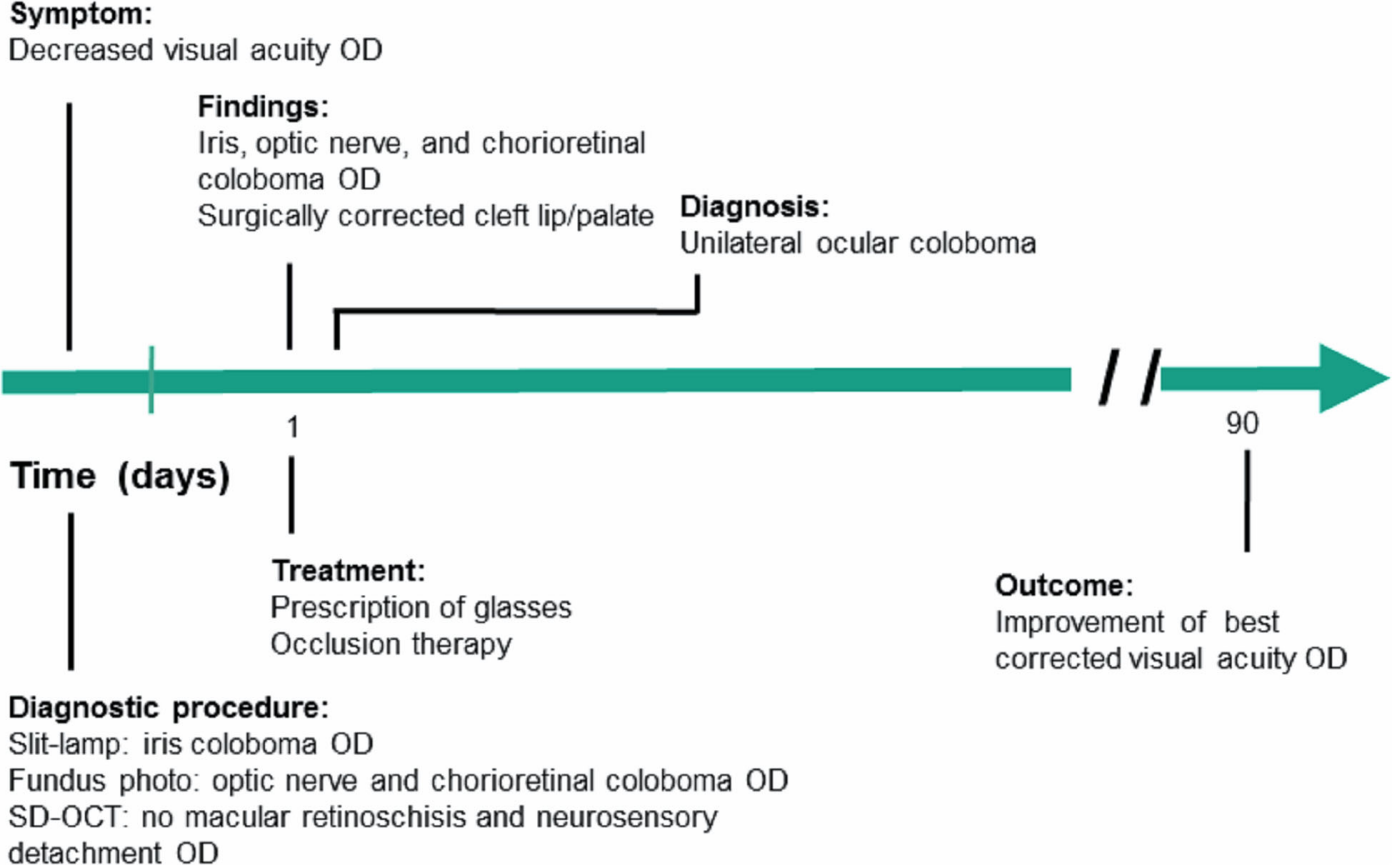
Clinical Timeline. A 5-year-old Asian boy diagnosed with unilateral ocular coloboma and cleft lip/palate without any other systemic abnormalities. OD: right eye, SD-OCT: spectral domain optical coherence tomography

**Fig. 2:**
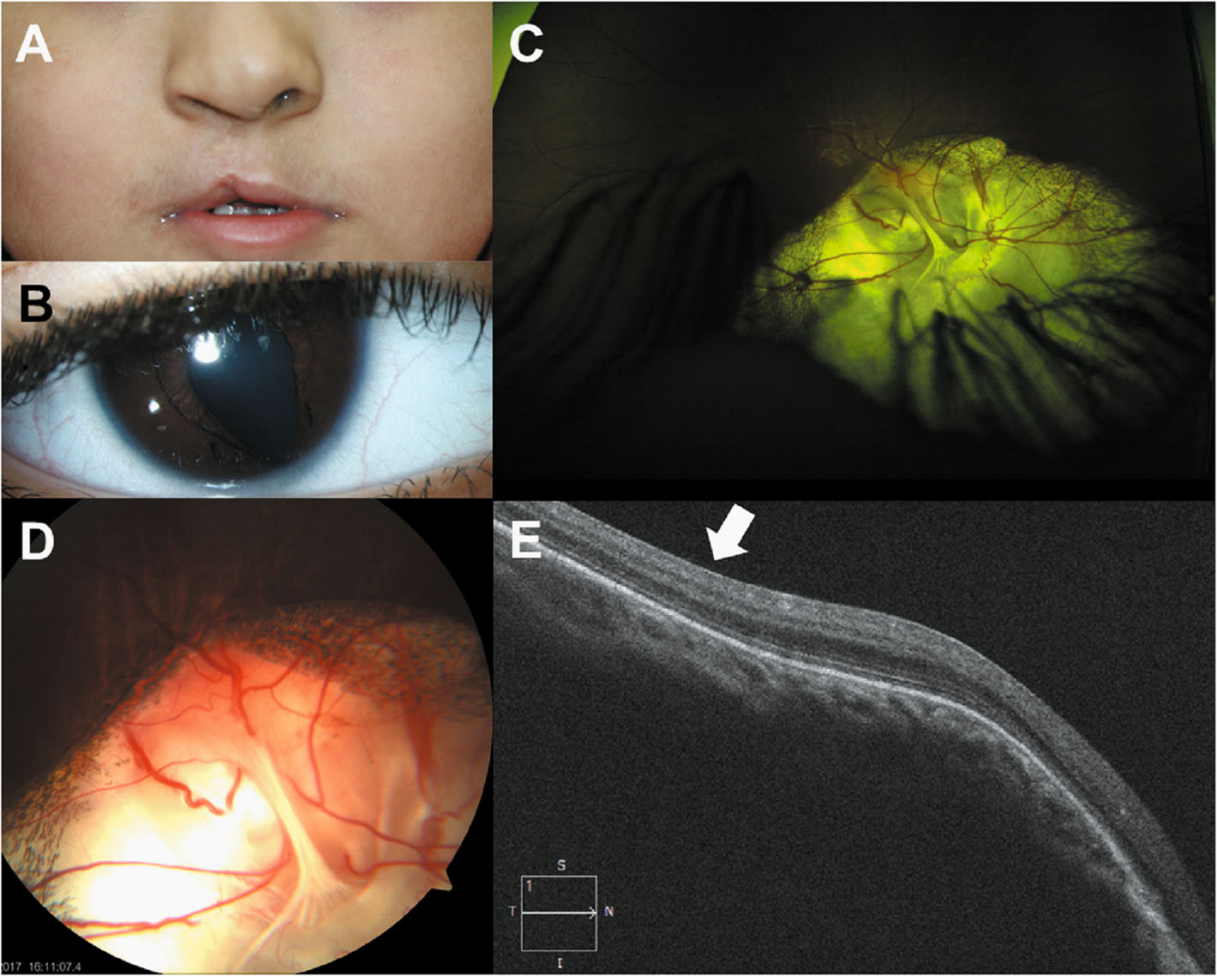
Clinical photographs of case. **a**, Face photograph showed surgically corrected unilateral cleft lip on the right side. **b**, Right iris coloboma in the typical inferonasal position. **c** and **d**, Fundus photograph of the right eye revealed coloboma of the optic disc, choroid and retina. **e**, Horizontal spectral domain optical coherence tomography images of the right eye showed preserved foveal anatomy without retinoschisis or neurosensory detachment

A 5-year-old Asian boy presented with decreased visual acuity in his right eye (Fig. 1). He was born with CL/P at 40 weeks of gestation. However, he had no family history of CL/P. His mother received a measles, mumps, and rubella vaccine during the first trimester of pregnancy. No relevant history of smoking, alcohol consumption, folate deficiency, exposure to ionizing radiation, or any severe infection during pregnancy was found. Prenatal fetal ultrasonography showed unilateral CL/P on the right side without brain lesions. Postnatal renal ultrasonography of the child revealed mild hydronephrosis in the right kidney without dysfunction. Follow-up examination at 4 years of age confirmed that both the kidneys were normal. He underwent several cardiological and endocrinological investigations for the evaluation of congenital rubella syndrome, which revealed no abnormal findings. Neurological examination revealed no midline defect of the vertebral bodies. At 9 months of age, he underwent successful surgical repair of his unilateral CL on the right side (Fig. 2a). He experienced two episodes of febrile seizures at the age of 2 years with no sequelae. Electroencephalography after the seizures revealed no remarkable findings. Although genetic analysis was recommended, his parents refused to undergo chromosomal evaluation. Magnetic resonance imaging showed no other midline defects or neurological anomalies. He did not have any intellectual or psychomotor developmental delays.

At initial presentation, his best-corrected visual acuity (BCVA) was 20/60 in the right eye and 20/25 in the left eye. Slit lamp examination revealed iris coloboma in the inferonasal quadrant of the right eye (Fig. 2b). Fundus examination showed optic nerve and chorioretinal colobomas in the inferonasal quadrant of the right eye (Fig. 2c and d). Spectral domain optical coherence tomography showed preserved foveal anatomy without retinoschisis or neurosensory detachment in the right eye (Fig. 2e). No evidence of microphthalmia, scleromalacia, congenital cataract, or ocular motility disorder was found. The left eye had no remarkable findings. He was prescribed glasses based on his cycloplegic refractive errors (Right eye: − 2.00 Dsph − 2.00 Dcyl × Axis 180°; Left eye: + 1.50 Dsph − 1.00 Dcyl × Axis 180°). We recommended daily occlusion therapy in the left eye for 4 h. After 3 months, BCVA in the right eye improved to 20/30. In addition, the patient was referred to the otolaryngology department for otological and audiological assessments, which revealed no relevant abnormalities.

## Discussion and conclusions

In our patient, unilateral ocular coloboma was associated with CL/P. However, no other neurological or systemic abnormalities were found. Previous studies have shown mutations in various genes associated with microphthalmia, anophthalmia, and coloboma (MAC) phenotypes [6]. However, none of these genes are considered a major causative gene of the MAC phenotype because each study found a definite mutation in only a small proportion of patients [6]. This reflects the heterogeneity of colobomatous deformities and the molecular complexity of ocular development. Our case revealed a unique association of unilateral ocular coloboma involving the iris, choroid, retina, and optic nerve with CL/P.

The co-existence of CL/P and ocular coloboma in the absence of other congenital malformations did not match any previously reported syndromes related to known genetic loci [6]. By the end of the 6th week of gestation, the upper lip and primary palate are completely formed; this occurs by the fusion of the medial nasal processes followed by merging with the maxillary processes of both sides [7]. Shortly before these fusions are completed, a peak in cell division activity of the lateral nasal process, rendering it susceptible to teratogenic insults, is observed [7]. At this critical time point, the closure mechanism may fail if events that prevent growth occur. Regarding normal eye development, the fusion of the optic fissure begins at the center at approximately 5th week of gestation and advances anteriorly along the rim of the optic cup and posteriorly along the optic stalk [8]. The underlying etiology of ocular coloboma is failure to close the ectodermal optic vesicle fissure [1]. Therefore, an insult during this period could result in the co-occurrence of these two anomalies.

Since rubella vaccine contains a live attenuated virus, which is a known teratogen, there might be concerns about fetal hazards occurring following vaccination during pregnancy [9]. However, prospective studies have revealed that rubella vaccination during pregnancy does not appear to affect pregnancy outcomes or cause congenital rubella syndrome [9]. We conducted a MEDLINE search for the association of maternal rubella vaccination with CL/P and found no case of CL/P associated with maternal rubella vaccination. A large-scale study involving six countries reported no association between CL/P and vaccination of unaware women in the early stages of pregnancy [10]. The potential risk factors of CL/P include maternal smoking [11], maternal alcohol consumption [12], nutritional factors such as folate deficiency [13], exposure to ionizing radiation and infection [7], and maternal obesity [7]. In our case, the patient’s mother confirmed that she was not exposed to any teratogenic risk factors during pregnancy.

CL/P and coloboma co-exist in several conditions, many of which are associated with other systemic defects. The genes associated with syndromic forms of coloboma tend to be widely expressed and generally have a pleiotropic effect [14]. CHARGE syndrome (coloboma, heart disease, choanal atresia, retardation of growth and development, genitourinary malformations, and ear abnormalities) is a rare genetic condition that occurs during early fetal development that affects multiple organ systems [15]. A partial deletion of the short arm of chromosome 4 is observed in patients with Wolf–Hirschhorn syndrome [16]. Wolf–Hirschhorn syndrome is characterized by microcephaly, intellectual impairment, Greek helmet facies, and closure abnormalities such as ocular coloboma and cardiac septal defect [17]. However, our patient did not meet the diagnostic criteria of CHARGE syndrome or Wolf–Hirschhorn syndrome and systemic defects were not suspected based on the patient’s diagnosis.

Our case is unique because extensive ocular coloboma and CL/P co-existed without other neurological abnormalities. Branchio-oculo-facial syndrome caused by *TFAP2A* gene mutations is a condition that acts on prenatal development, especially the structures of the face and neck [4]. Features of this syndrome include skin abnormalities on the neck, eye and ear anomalies, and characteristic facial aspects [4]. However, in our case, no skin lesions such as brachial arches or characteristic facial deformities were noted. A case of CL and choroidal coloboma associated with endocrine abnormalities due to hypothalamic-pituitary dysfunction has been previously reported [5]. However, in our case, ocular coloboma was more extensive than previous reported cases and no endocrine abnormality associated with midline defects was noted.

Visual acuity in children with optic nerve coloboma is determined by the preservation of normal foveal anatomy [9]. The size of coloboma, optic nerve color change, or the presence of foveal pigmentation has no significant effect on central visual acuity [18]. Although significant refractive errors and anisometropia are common in patients with optic nerve coloboma, the most critical factor associated with good vision is the extent of foveal involvement of the coloboma [18]. Fortunately, preserved foveal structure in our patient led to a relatively good visual prognosis after correction with glasses.

In conclusion, we reported a case of ocular coloboma combined with CL/P alone. This case indicates that in a patient with CL/P, attention should be paid to the possibility of ocular coloboma even in the absence of other systemic or neurological abnormalities.

## Data Availability

All data generated or analysed during this study are included in this published article.

## Abbreviations

BCVA: Best-corrected visual acuity
CL/P: Cleft lip/palate
MAC: Microphthalmia, anophthalmia and coloboma
